# Editorial: Advances in the application of technology for monitoring horse welfare and health

**DOI:** 10.3389/fvets.2025.1715133

**Published:** 2025-10-16

**Authors:** Emanuela Dalla Costa, Marco Bovo

**Affiliations:** ^1^Department of Veterinary Medicine and Animal Sciences, University of Milan, Lodi, Italy; ^2^Department of Agricultural and Food Sciences, University of Bologna, Bologna, Italy

**Keywords:** equine monitoring, wearable sensors, computer vision, horse behavior, PLF

## Abstract

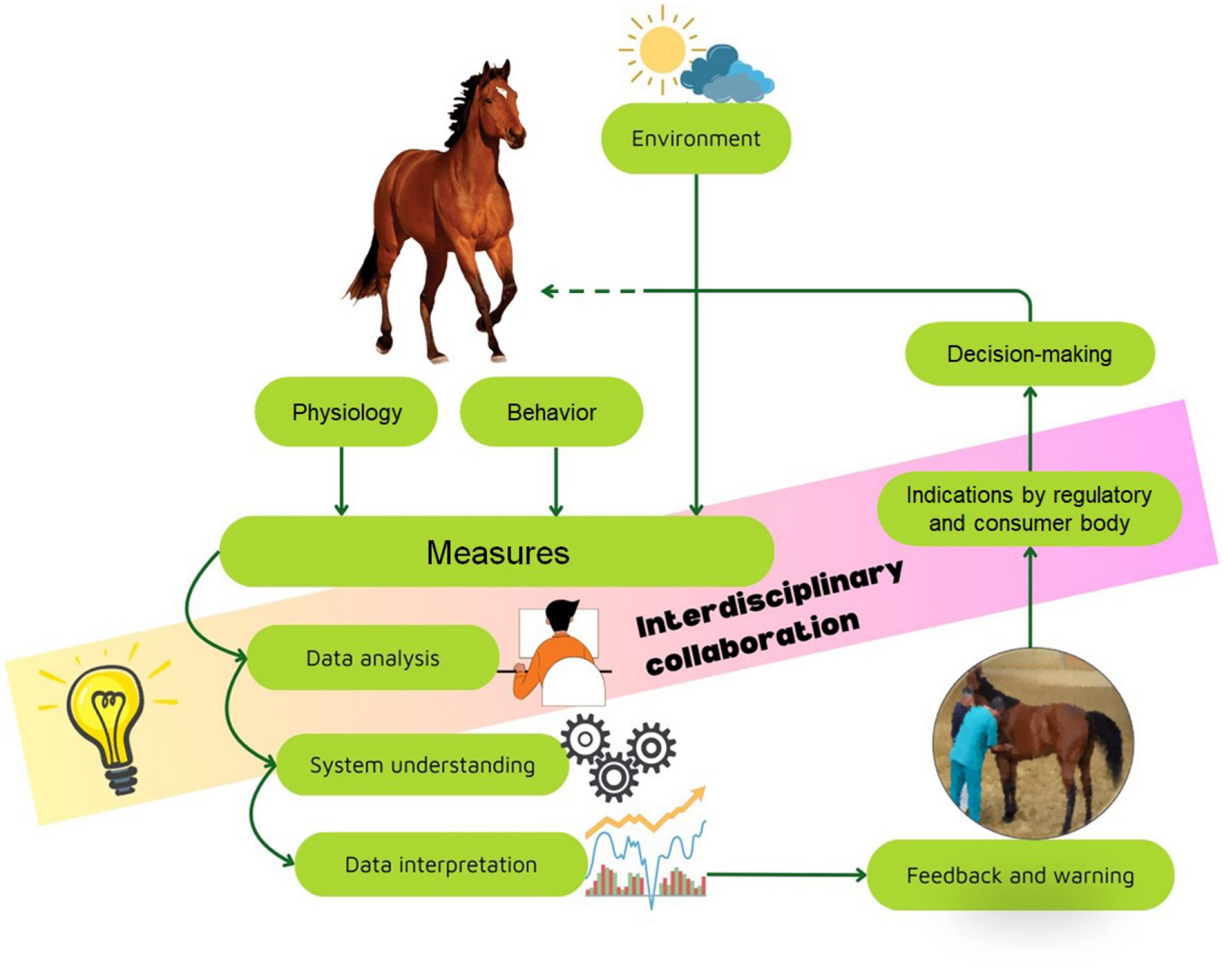

The dynamic role of horses in today's society, from sports and leisure riding to therapeutic activities and companionship, underscores the critical need for effective monitoring of their health and welfare. Horses are highly sensitive animals, and, similar to other species, their wellbeing has a direct impact on their performance, longevity, and quality of life. In recent years, animal welfare has become a topic of increasing interest, evolving into an issue of scientific, regulatory, and ethical significance. This has stimulated intense research and development in various areas, including laboratories, farms, zoos, and the care of companion and working animals. This increased attention reflects a growing awareness of the need to improve the living conditions of animals while promoting environmental sustainability (1–4). In equines, welfare assessment is still a challenge, given the complexity of integrating multiple indicators into a representative framework (5–7).

To date, equine welfare is assessed through direct observation of animal-based measures, such as body condition or the presence of injuries along with physiological indicators such as blood cortisol concentrations. However, these approaches have significant limitations, including the time needed for the assessment, the necessity of training new assessors to ensure reliable evaluations, and the inability to ensure continuous monitoring (8, 9).

These limitations have led to an increasing exploration of innovative technologies for monitoring equine welfare. Advances in livestock farming technologies, including wearables, environmental sensors, and computer vision, could transform the management practices for domestic animals, including horses. These technologies could provide not only real-time health monitoring but also early detection of anomalies that may indicate disease, which would be invaluable for research, veterinary care, and horse owners alike. While several promising technologies, such as accelerometers, pressure sensors, and video surveillance, have demonstrated high potential in research settings, many have yet to transition successfully into practical, everyday applications. In fact, the majority of these technologies have been tested and validated for research purposes, but few of them are, to date, applicable to daily management. In an operational environment, these technologies still face some limitations (3, 8). Thus, at least for now, the great theoretical potential of these new technologies has had little impact on reality.

Within this context, the present editorial summarizes the main contributions collected in this Research Topic, which aims to explore the evolution, validation, and on-the-ground application of technology for monitoring horse health and welfare (see Graphical Abstract). This Research Topic consists of a collection of ten studies that deal with advancing the integration of new technologies into routine use. This in order to ensure that new technologies meet the high standards required for accuracy and consistency, so bridging the gap between experimental research and real-world application. In the context of precision farming, wearable technologies are among the most widely studied innovations for monitoring equine welfare. These devices are typically fitted to harnesses, girths, or collars, and are designed to capture continuous physiological and biomechanical data. Accelerometers, gyroscopes, and GPS sensors record locomotion, gait patterns, and workload intensity, allowing trainers to tailor exercise regimens to individual horses. Such systems are critical for detecting subtle asymmetries or irregularities that may signal early lameness. Lameness remains one of the most pressing welfare concerns in horses, often resulting in a reduced quality of life and compromised performance. Technological advances in monitoring enable the automated detection and modification of gait abnormalities. Three studies investigated horse gaits and the relation to morphological aspects and work tasks (Zupan Šemrov et al.), kinematic modifications and training fatigue (Siegers et al.) and inertial measures for different breeds (Asti et al.). The ability to monitor changes in daily activity patterns provides valuable insight into welfare indicators such as stress, discomfort, or illness. Connected to the need to evaluate the effects of training sessions are the studies by Wonghanchao et al., monitoring the stress response via heart rate variability (HRV) during consecutive days of jumping competitions, and Garcia Carvalho et al., evaluating the response to a single whole-body vibration session as a recovery measure. The results provided in these articles highlight the need to be mindful of potential stress that could, at least in part, impact the welfare of horses participating in close-time competitions and propose robust methodologies to estimate it. In addition, Itoh et al. proposed and applied a novel method for non-invasively recording multichannel electroencephalography with the aim of demonstrating the feasibility and validity of this approach for investigating brain function in horses, paving the way for further applications, such as examining cognitive abilities or brain disorders in horses. The objective of the manuscript published by Tucker et al. was to apply computational fluid dynamics analysis to an equine head inhalation model that replicates recurrent laryngeal neuropathy (RLN): the authors evaluated fluid dynamics impedance for four surgical procedures. Troillet and Scharner provided a detailed description and exhaustive documentation of a cohort of five horses that were successfully treated with an incomplete bypass procedure, demonstrating positive long-term outcomes and the advancements in surgical techniques by implementing the closure of the mesenteric gap.

Technologies are now also integrated into equine living environments to improve welfare. Stable-based sensors monitor critical air quality parameters such as temperature, humidity, and ammonia concentration, which are critical for respiratory health. Automated surveillance cameras, coupled with AI-based behavior recognition, can detect stereotypic behaviors such as cribbing or weaving, which often signal stress or poor welfare. IoT frameworks that combine individual wearables with environmental sensors create holistic welfare assessments at both the individual and group levels (10). In this dynamic context, Gobbo et al. explored the methods to assess positive horse behavior in relation to their environment to provide information that enhances animal welfare. With the adoption of accelerometers, the authors achieved a non-invasive continuous monitoring of lying behavior, thus suggesting a way to continuously monitor horse behavior in relation to their management and housing. Finally, in a recent article, Velineni et al. tested a new glucometer and reported that this new generation of tools represents an improvement over its predecessor and represents a reliable and cost-effective method for accurately monitoring blood glucose levels in horses in farm, clinic, or laboratory environments.
